# Social–Emotional Functioning and Quality of Life in Language Disorders: A Systematic Review of Development From Childhood to Adolescence

**DOI:** 10.1111/1460-6984.70039

**Published:** 2025-04-22

**Authors:** Mélanie van Barreveld, Annette Scheper, Constance Vissers, Iris Duinmeijer, Britt Hakvoort

**Affiliations:** ^1^ Behavioral Science Institute Radboud University Nijmegen the Netherlands; ^2^ Research Department Royal Kentalis Utrecht the Netherlands; ^3^ Research & Development Department NSDSK Amsterdam the Netherlands; ^4^ Research Department Royal Dutch Auris Group Rotterdam the Netherlands

**Keywords:** developmental language impairment, longitudinal, quality of life, social–emotional functioning

## Abstract

**Background:**

It is well‐established that children and adolescents with developmental language disorder (DLD) have social–emotional difficulties. This is reflected in their behaviour, for instance, by (social) withdrawal, hyperactivity or difficulty with peer relations. Children with DLD are also known to have poorer quality of life (QoL). This is likely to be related to social–emotional difficulties, for both concern similar developmental domains but from a different point of view. Findings on the social–emotional abilities, QoL and predictors thereof in children with DLD are inconsistent across studies.

**Aims:**

This review investigates how social–emotional functioning (SEF) and QoL develop from childhood into early adulthood in children with DLD. These developments are then compared and predictors are identified.

**Methods & Procedures:**

A systematic review of 128 articles, conducted following the Preferred Reporting Items for Systematic Reviews and Meta‐Analysis guidelines in January 2024, yielded 34 articles for inclusion after qualitative assessment. Clinical populations were labelled differently (e.g., DLD, specific language impairment (SLI), language impairment (LI)) but adhered to inclusion criteria for language disorder (LD). The majority of the articles focused on SEF (*n* = 30 articles), while the remaining examined QoL (*n* = 4 articles).

**Main Contribution:**

This is the first review to simultaneously investigate SEF and QoL in children with LD. No single developmental pattern was found for SEF: a range of possible developmental trajectories exists. Interestingly, prosocial skills generally appear to improve over time, whereas children also experience increasing problems with peer relations. Few studies employed a longitudinal design regarding QoL, but those that did suggest that children with LD are likely to have poorer and declining QoL, at least between the ages of 4 and 9. The sole study examining SEF and QoL in the same sample found a predictive relationship between early SEF and later QoL. Linguistic abilities were predictive in less than half of the studies on SEF development and had little impact on QoL development. Findings on other predictors were inconsistent.

**Conclusions:**

Despite their interrelatedness, SEF and QoL do not necessarily develop similarly in children with LD. Only one study examined SEF and QoL in the same children and found contrasting developmental trends. This could imply that SEF and QoL are not as intertwined as presumed. It also remains unclear what best predicts change over time in these two dimensions. More research is necessary to further examine the relationship between SEF and QoL, as well as to identify potential predictors.

**WHAT THIS PAPER ADDS:**

*What is already known on the subject*

- Children with LD are more likely than their peers without LD to have lower SEF and poorer QoL. However, the development of these domains across childhood and adolescence remains unclear. The relationship between SEF and QoL is equally under‐researched.

*What this paper adds to the existing knowledge*

- This is the first review on those with LD that takes a longitudinal perspective on both SEF and QoL and looks at their relationship. It highlights that longitudinal research is valuable and necessary, specifically for QoL, where studies are scarce. Only one study examined SEF and QoL in the same group of children and found a predictive relationship between (aspects of) SEF and later QoL.

*What are the potential or actual clinical implications of this work?*

- This study highlights the relevance of longitudinal research when aiming to comprehend development, particularly in heterogeneous populations such as language disorders (LD). Clinicians are advised to address social–emotional problems alongside language to potentially increase SEF and QoL. Future research should investigate SEF and QoL simultaneously to substantiate the preliminary evidence for this relationship between SEF and QoL. Additionally, future studies consider support and multilingualism as potential predictors of this development in children with LD.

## 1 Introduction

Language and communication are essential to our daily lives and society. Many children seem to acquire language abilities rather easily, but some children's language acquisition falters. Approximately 5–7% of young children have developmental language disorder (DLD), which is primarily characterized by problems with language acquisition (Bishop et al. 2017). Difficulties with language and the development thereof can have widespread consequences for children's general development. Children with DLD are known to show, amongst others, difficulties to form relationships, understand emotions, and control impulses (e.g., Durkin and Conti‐Ramsden 2010; Goh et al. 2021). Such abilities are what this review refers to as social–emotional functioning (SEF).

Language development is rooted in the social interactions and linguistic resources in the child's environment. Language's function is crucial, as speech mediates between the child's internal (i.e., emotional) and external (i.e., social) world, and through meaning children come to understand these two worlds (Vygotsky 1934). Social and emotional functioning are therefore intertwined: by developing social skills in interaction, children come to understand and manage their emotions. Children's SEF grows through interaction with their environment (Sameroff 1975). Vygotsky's so‐called interactionist theory also posits that language and social interactions are a prerequisite for the development of cognitive functions such as executive functions (EF) and theory of mind (ToM).

Following from the relationship between language, cognition and behaviour, children with DLD have problems in SEF (Charman et al. 2015; Conti‐Ramsden et al. 2019; Law et al. 2009; Le et al. 2020). More recently, the quality of life (QoL) of children with language disorders (LD) has received more attention, as this is also often negatively affected in this group (Feeney et al. 2017; Le et al. 2020). QoL is a multifactorial construct defined as ‘an individual's perception of their physical, psychological and social well‐being’ (Eadie et al. 2018: 800). This concept thus concerns perceptions of life areas, and SEF involves specific abilities that can be part of these life areas. An individual's social–emotional abilities are therefore closely related to their experienced QoL. Longitudinal designs could aid in understanding this variation, as they track progression over time, allowing for the identification of causal relationships and factors contributing to resilience or vulnerability in development. It is crucial to shed more light on what longitudinal studies on the development of SEF and QoL in children with DLD teach us about this group.

### 1.1 Social–Emotional Functioning in DLD

**FIGURE 1:**
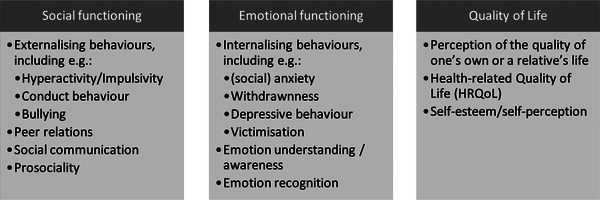
Overview of the terms used that refer to social functioning, emotional functioning and quality of life in this review.

SEF and the development thereof have received much attention in research on children with DLD. Across studies, different ways of framing social–emotional difficulties have been used, such as the internalizing–externalizing divide. Internalizing behaviours are negative behaviours focused inward (e.g., anxiety, worry, (social) withdrawal), whereas externalizing behaviours are directed toward others (e.g., hyperactivity, aggression, bullying). In reality, it is hard to tease social and emotional functioning apart, as their development is intertwined from an early age. For the purpose of this review and its focus on social and emotional functioning, we will distinguish between problems in the social domain and those within the emotional domain (Figure 1). Issues in the social domain closely relate to externalizing difficulties and concern relations and communication with others (e.g., family, peers, teachers), prosociality (i.e., responding positively to others’ needs and welfare; Toseeb et al. 2017), conduct problems (i.e., violations of rules and social norms through behaviour), impulsivity and hyperactivity. Like conduct problems, impulsive and hyperactive behaviour can affect social communication, hence we assign them to the social domain. Difficulties in the emotional domain are internalizing characteristics, such as (social) anxiety, and weak emotional understanding and recognition. Note that the relationship between social and emotional problems is reciprocal: more social problems are likely to result in, for example, anxiety or depressive symptoms, and problems with mentalization or emotion regulation can lead to challenges in social communication. Children might, for instance, interpret others’ emotions inadequately or be unable to regulate their own emotions, which can lead to inappropriate behaviour in response to the social situation.

Communication difficulties and social–emotional problems often occur together. The inability to properly communicate can lead to feelings of frustration or insecurity, which might result in, for example, aggressive, inattentive, withdrawn or anxious behaviours. Results of studies investigating the effect of early language difficulties on later SEF in DLD confirm this association (Yew and O'Kearney 2015; 2017). Some studies also indicate that children with more severe language difficulties might experience elevated levels of social–emotional problems (Chow et al. 2018; Clegg et al. 2015; Lindsay and Dockrell 2012). However, the knife might cut both ways: social–emotional skills can also impact the development of language and communication abilities. Children exhibiting withdrawn, inattentive or aggressive behaviour may face challenges in social situations. They are less favoured as communicative partners, or their behaviour complicates interaction with others (Chen et al. 2020). Both language comprehension and production are crucial for interactive alignment, as they are both used to predict the dialogue and make it flow (for more detail, see Garrod and Pickering 2009, 2013; Pickering and Garrod 2014). No investigations of the effect of these behaviours on language in children with DLD are known to the authors. However, for children with TD, studies confirm the bidirectionality of the association between language and SEF (Girard et al. 2016; Salmon et al. 2016; Wang et al. 2018).

Numerous studies confirm elevated levels of social–emotional difficulties in children with DLD compared with TD peers (Charman et al. 2015; Goh et al. 2021; Levickis et al. 2018; St Clair et al. 2011; Duinmeijer et al., submitted). Problems are found in all domains, but difficulties with peer relations are the most prevalent. Differences between children with DLD and their TD peers can be apparent as early as the preschool age (McCabe 2005). Still, the occurrence and severity of problems vary across and within the social and emotional domain as well as across ages (Curtis et al. 2018). The relatively limited use of longitudinal designs could explain the mixed results. Many studies use either cross‐sectional designs or assess children at a specific age, rather than following the same children over time.

### 1.2 Quality of Life in DLD

The body of research investigating the impact of DLD on QoL has grown due to increased awareness of the lifelong consequences of DLD. QoL is a broad construct, which engages in the perspective of the individual on a variety of life areas. Questionnaires on QoL typically provide an overarching score, as well as scores on several domains. The most commonly used questionnaires include questions on physical, social, emotional and school functioning (e.g., PedsQL, TAPQOL, KINDL). At first glance, QoL might appear very similar, if not overlapping, with SEF. Indeed, the two constructs are related, as they question similar aspects, albeit with a different approach. Questionnaires looking into SEF are skill‐oriented, whereas QoL questions on social and emotional functioning ask the participant to evaluate their daily functioning on a variety of areas, including SEF.

In general, parents of children and adolescents with DLD report reduced QoL compared with typical peers (see Le et al. 2020 for an overview). Findings on the specific QoL domains diverge depending on age and study sample, but poorer QoL in any of these domains can be expected based on the broad impact of the disorder. Poor social and/or emotional QoL can be a result of poorer SEF but can also occur together with average SEF scores. Lower QoL in the physical and school functioning domains might also be reported in children with DLD. Yet, poorer physical functioning is not often thought of as an area that is typically affected in DLD. In reality, daily events such as going to school and playing with others can be more strenuous for children with DLD due to the high demands on language and communication. This might lead to headaches, stomach aches and/or fatigue. The academic achievement of children with DLD is known to be affected due to the central role of language in education. Lower educational outcomes are reported for DLD compared with typical development (Law et al. 2009). Reduced QoL scores in this domain are therefore unsurprising. Unfortunately, there has been very little research into QoL in this group, and most studies either assessed children at a specific age or looked at QoL cross‐sectionally.

### 1.3 Aims

SEF and QoL are clearly at risk for those with DLD. The purpose of this review is to gain more insight into the development of SEF and QoL in children with DLD and the potential predictors of changes in this development. Longitudinal designs can help to unravel the variation across studies that assess SEF or QoL solely at one moment in time or across age groups. Considering that DLD does not resolve in adolescence or adulthood, a thorough understanding of the manifestation of both good and poor SEF and QoL in this clinical group is necessary to provide adequate care and support. This knowledge can give impetus to our understanding of the development of children with DLD and potential critical ages. Our focus will therefore be the period from childhood to early adulthood (about 25 years). This review will also provide direction to the focus points of future studies. We will address the following research questions:

- What do longitudinal studies tell us about the development of SEF and QoL in children and adolescents with language disorder (LD)?
- Which factors predict development in SEF and QoL?
- Do SEF and QoL develop qualitatively similar, that is, does QoL improve when SEF improves and vice versa.

Based on the literature discussed here, we expect that (1) children and adolescents with LD have persistent difficulties in (at least some) aspects of SEF and QoL, (2) the severity of LD contributes negatively to SEF and QoL, and (3) SEF and QoL show comparable trends over time, as we know that the two are related with respect to the life areas they address.

## 2 Method

This systematic review adopted the Preferred Reporting Items for Systematic Reviews and Meta‐Analysis protocol (PRISMA; Page et al. 2021).

### 2.1 Search Strategies

**FIGURE 2:**
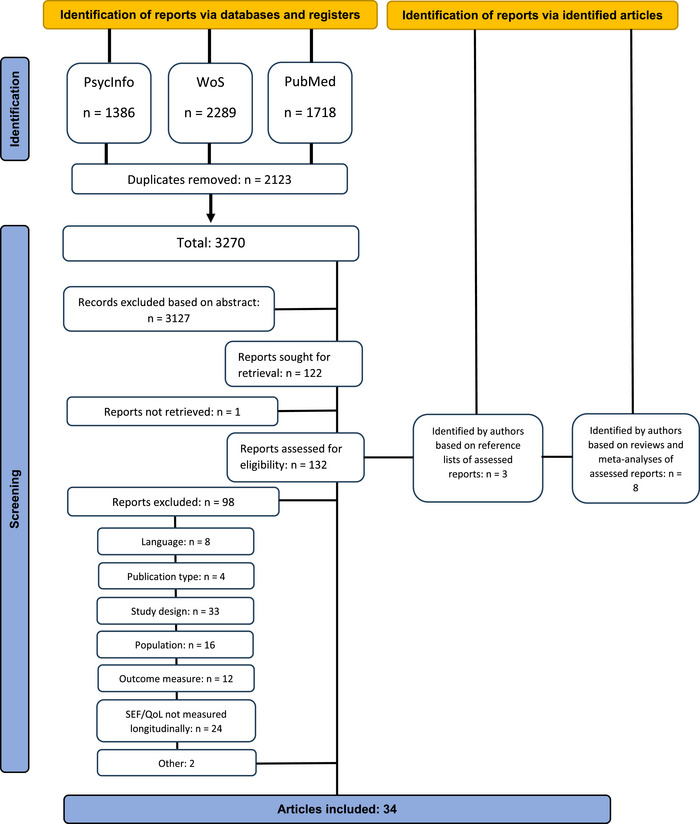
PRISMA flow chart.

Web of Science, PubMed and PsycInfo (Ovid) databases were consulted from 2000 to 2023. The first author carried out the search. Search terms were created in threefold. The first set of search terms aimed to identify DLD research, and another extensive list of terms was designed to locate literature on SEF and QoL, including synonyms and terms referring to specific aspects of SEF and QoL. Terms to identify children and adolescents made up the third set of search terms. Appendix A includes the full set of search terms. These three sets were combined into one search query, with the Boolean operators ‘OR’ distinguishing between terms within a set and ‘AND’ to separate the three sets. In PsycInfo we filtered for peer‐reviewed articles. The other databases solely include peer‐reviewed articles by default. An initial database search yielded 5393 publications, of which 2123 were duplicates. The full process is presented in Figure 2.

### 2.2 Eligibility Criteria

To be included in this review, studies were required to meet the following inclusion criteria:

- The main topic of investigation is any aspect of SEF or QoL in children or adolescents with DLD (see search query for included equivalents).
- The diagnosis of LD meets one of the following criteria:
  ◦ A score of −1.25 standard deviations (SD) below the norm on a single standardized language (sub)test.
  ◦ A score of 1 SD below the norm on multiple standardized language (sub)tests or a composite score.
  ◦ A diagnosis of a LD (such as DLD, specific language impairment (SLI), or language impairment (LI)) made by a qualified professional. Diagnoses based solely on parent or teacher reports are insufficient.
- Experimental studies are quantitative, original and longitudinal. A study was considered longitudinal when it included at least two measurements of the same aspect of SEF and/or QoL.
- Reviews need to include quantitative, longitudinal studies.
- The maximum age of participants at the first follow‐up is 25 years.
- The full article is available through open access or Radboud University.

### 2.3 Data Collection

Initial screening of the titles and abstracts was primarily done by the first author, who was doubled by one of the other authors. This was done using the Rayyan program. Cases of doubt were discussed among the authors. Based on this screening, 3127 articles were excluded because at least one of the inclusion criteria was disregarded. The first author extracted the data of the remaining 128 using both Rayyan and manual extraction. The extracted information included, amongst others, author(s), year of publication, country, sample characteristics, the cohort the study reported on (if applicable), materials and results. These 128 publications were fully read and reviewed by the first author. To evaluate the reliability of exclusion/inclusion decisions, all articles were read and reviewed by at least one other reviewer. Articles on which reviewers did not converge were discussed to reach agreement. After this second review, 34 articles were included in this study.

### 2.4 Quality Assessment

The quality of the included studies was assessed by all authors using the Kmet et al. (2004) quality checklist for quantitative studies, which includes 14 criteria for evaluating studies’ internal validity. As the studies included here are longitudinal, three criteria were not applicable. Raters attributed scores ranging between 0 and 2 to each criterion using the scoring manual. The first author completed the quality assessment for all articles and was doubled on 10% of the articles by one of the other authors to check interrater agreement. All studies had a quality rating above 75% (the ‘conservative’ cut‐off point according to Kmet et al. (2004)) and agreement on inclusion/exclusion was 100% despite some differences in scores on specific criteria. This means no articles were excluded based on the quality assessment.

## 3 Results

### 3.1 Description of Included Studies and Risk of Bias

Out of the 34 included studies, the majority investigated (an aspect of) SEF (30/88%), and 4 studied QoL (10%). One of the studies investigated both SEF and QoL but is classified under QoL for matters of clarity. Of the studies on SEF, 3 were published before 2010, 17 between 2010 and 2019, and 10 were published later than 2019. The QoL articles were published relatively recently, with 3 between 2010 and 2019 and 1 after 2019.

Table 1 provides an overview of the included studies' characteristics. There is considerable variation in the terminology used to describe the studied clinical population. In the remainder of this review, LD will be used to refer to the clinical population to generalize results across articles. When specific articles are discussed, we will adhere to their chosen definition of their studied clinical group (amongst others: DLD, rDLD, SLI, LI, LL). Each article studied clinical populations, and their definitions are given in Table 1.

**TABLE 1:** Participant characteristics and risk of bias of reviewed literature.^a^

Study	Country	Clinical group	Diagnostic criteria	Comparison group	Time points (age, years)	*n* clinical group	*n* comparison group	Attrition rate clinical group
Bao et al. (2016)	Canada	Speech and/or language disorder	−1 SD on PPVT or spoken component of TOLD OR −2 SD on any subtest on TOLD, OR −2 SD on the content and sequence subtests of the GFWCT‐AM	TD	5	103	142	
					19	85	135	18.5%
					25	74	132	28.2%
					31	68	123	34%
Botting et al. (2016)	UK	Language impairment (LI)	Attending language unit in the UK and identified with primary speech and language difficulties without neurological difficulties, hearing impairment, autism or general learning disability	AMP	7	242	×	
					16	139	121	42.6%
					17	90	90	62.9%
					24	84	66	65.3%
Botting and Conti‐Ramsden (2000)	UK	Specific Language Impairment (SLI)	Attending language unit in the UK and identified with primary speech and language difficulties without neurological difficulties, hearing impairment, autism or general learning disability	×	7	242	×	
					8	234		3.4%
					4			
Conti‐Ramsden et al. (2019)	UK	SLI	Attending language unit in the UK and identified with primary speech and language difficulties without neurological difficulties, hearing impairment, autism or general learning disability	×	7	242	×	
					8	232		4.2%
					11	200		17.4%
					16	139		42.6%
Durkin et al. (2017)	UK	LI	Attending language unit in the UK and identified with primary speech and language difficulties without neurological difficulties, hearing impairment, autism or general learning disability	AMP	17	90	91	
					24	84	88	6.7%
Eadie et al. (2018)	Australia	DLD	DLD: > 1.25 SD below the mean on the CELF receptive and/or expressive scales at both 4 and 7 years. Severe DLD: > 2 SD below the mean on the CELF receptive and/or expressive scales at 7 years. Mild/moderate DLD: between 1.25 and 2 SD below the mean.	TD	4	70	802	Only participants with complete data were included
					7			
					9			
Forrest et al. (2020)	UK	Risk of DLD (rDLD)	Risk of DLD: (1) language developing slowly and/or doesn't understand others; (2) 1.5 SD below norm on British Ability Scales naming test. No additional diagnoses.	General population (GP)	3	891	13 371	Unclear how many participants had data at each measurement due to imputation
					5			
					7			
					11			
					14			
Forrest et al. (2018)	UK	rDLD	Risk of DLD: (1) language developing slowly and/or doesn't understand others; (2) 1.5 SD below norm on British Ability Scales naming test. No additional diagnoses	GP	3	891	13 371	Unclear how many participants had data at each measurement due to imputation
					5			
					7			
					11			
					14			
Goh et al. (2021)	UK	DLD	At least −1.5 SD or below on 2/6 language composite scores (vocabulary, grammar, narrative, receptive and expressive language). No comorbid disorders	Typical language (TL)	4–5	91	392	Only participants with complete data were included
					7–9	91	392	
Howlin, Mawhood, and Rutter (2000)	UK	LI	Attending school for severe language disorders and/or autism	ASD	4–9	23	19	
					24	20	19	13%
Jelen et al. (2023)	UK	LD	At least –1.5 SD or below on 2/6 language composite scores (vocabulary, grammar, narrative, receptive and expressive language). Inclusion comorbid disorders	No LD	5–6	137	392	
					10–11	80	270	41.7%
					10–11 | parents	31	154	
					12–13	50	183	63.6%
					12–13 | parents	26	124	
Le et al. (2021)	Australia	Low Language (LL)	1.25 SD or more below population mean on a the CELF‐4 or ‐P2 core score. No exclusion of children with comorbid disorders	TL	4	160	1322	
					5	70	818	56.3%
					6	71	826	55.7%
					7	109	964	31.9%
					9	95	919	40.6%
					11	44	767	72.5%
					13	33	674	79.4%
Levickis et al. (2018)	Australia	Language disorder (LD)	1.25 SD or more below the mean on expressive and/or receptive language score	No LD	4	102	669	NA because LD was reassessed at every measurement
					5	88	683	
					7	122	649	
Lindsay and Dockrell (2012)	UK	SLI	Teacher identification followed by language assessment with TROG, PPVT, Bus Story & BAS scales	×	8	69	×	
					10	65		5.8%
					12	64		7.2%
					16	55–65		20.3% to 5.8%
					17	54		21.%
Lindsay et al. (2010)	UK	SLI	Vocabulary, grammar and expressive narrative scores below NVIQ. Exclusion of children with e.g. autism	×	5	69	×	
					16	54		21.1%
					17	54		
Mok et al. (2014)	UK	SLI	Attending language unit in the UK and identified with primary speech and language difficulties without neurological difficulties, hearing impairment, autism or general learning disability	×	7	242	×	
					8	232		4.2%
					11	200		17.4%
					14	113		53%
					16	139		43%
Pickles et al. (2016)	UK	LI	Attending language unit in the UK and identified with primary speech and language difficulties without neurological difficulties, hearing impairment, autism or general learning disability	×	7	164	×	Only participants with complete behavioural data on 3/4 measurements were included
					8			
					11			
					16			
Redmond and Rice (2002)	USA	SLI	Language impairment in preschool. Criteria: (1) identified as language‐impaired by SL pathologist, (2) performance on PPVT‐R > 1 SD below mean, (3) expressive language > 1 SD below age expectations (MLU), (4) normal intellectual functioning, (5) pass on articulation test, (6) normal hearing	AMP	5			No attrition
					6–7			
					7–8	12	17	
Roy and Chiat (2014)	UK	SLI	Referral to SLT services. No report of congenital problems, hearing loss, oro‐motor difficulties, ASD, no concerns about NVIQ, first language English	×	2;6–4	91	×	No attrition. Not that speech–language problems were redefined at T3, leaving *n* = 53 children with deficit(s) at T3
					4–5			
					9–11			
Samson et al. (2020)	NL	DLD	Formal DLD diagnosis following the DSM‐IV criteria for language impairment: receptive or expressive problems are at least 2 SD below the mean on a general measure or 1.5 SD below the mean on 2/4 language areas	TD	9–16	104		
					T1 + 9 months	90		13.5%
St Clair et al. (2019)	UK	rDLD	Risk of DLD: (1) language developing slowly and/or doesn't understand others; (2) 1.5 SD below norm on British Ability Scales naming test. No additional diagnoses	GP	3	884	13 344	Unclear due to imputation
					5			
					7			
					11			
St Clair et al. (2011)	UK	SLI	Attending language unit in the UK and identified with primary speech and language difficulties without neurological difficulties, hearing impairment, autism or general learning disability	×	7	234	×	
					8	203		13.2%
					11	167		28.6%
					16	103		66%
Toseeb et al. (2023)	UK	DLD	At least two of the following criteria: (1) pragmatic language > 1 SD below standardized mean (CCC‐2‐NL), (2) NWRT > 1 SD below standardized mean, (3) receptive language > 1 SD below standardized mean, or (4) positive response to ‘child has ever had speech/language therapy’	No DLD	Starting sample	534	5099	
					7 | *n* = 477	477	5009	10.7%
					9 | *n* = 507	507	5152	5.1%
					11 | *n* = 448	448	4784	16.1%
					13 | *n* = 430	430	4604	19.5%
					16 | *n* = 332	332	3861	37.8%
Toseeb et al. (2020)	UK	DLD	At least two of the following criteria: (1) pragmatic language > 1 SD below standardized mean (CCC‐2‐NL), (2) NWRT > 1 SD below standardized mean, (3) receptive language > 1 SD below standardized mean, or (4) positive response to ‘child has ever had speech/language therapy’	No DLD	< 2	394	6137	Unclear due to imputation.
					7–9			
					11			
Toseeb et al. (2017)	UK	LI	Attending language unit in the UK and identified with primary speech and language difficulties without neurological difficulties, hearing impairment, autism or general learning disability	AMP	11	130	×	
					16	126	65	3.1%
					24	84	64	35.3%
Toseeb and St Clair (2020)	UK	r DLD	Risk of DLD: (1) language developing slowly and/or doesn't understand others; (2) 1.5 SD below norm on British Ability Scales naming test. No additional diagnoses	GP	5	738	12 972	Unclear due to imputation
					7			
					11			
Tsou et al. (2023)	NL	DLD	Recruited at special treatment groups, all formally diagnosed with DLD (> 1.5 SD below average on standardized language test)	No DLD	3;6	92	143	
					5;8	71	85	22.8%
Van den Bedem (2020)	NL	DLD	Diagnosis according to DSM‐V. No hearing impairment or ASD	No DLD	9;2‐16;3 | *n* = 98	98	156	
					T1 + 9 months | *n* = ?	70	155	28.6%
					T2 + 9 months | *n* = ?	62	132	36.7%
Van den Bedem et al. (2019)	NL	DLD	Diagnosis according to DSM‐V. No hearing impairment or ASD	No DLD	8;4–16 | *n* = 114	114	211	
					T1 + 9 months	104	181	8.9%
					T2 + 9 months	99	155	13.2%
Van Den Bedem et al. (2018a)	NL	DLD	Diagnosis according to DSM‐V. No hearing impairment or ASD	No DLD	8;5–16	112	214	
					T1 + 9 months	104	183	7.1%
					T2 + 9 months	98	156	12.5%
Van den Bedem et al (2018b)	NL	DLD	Diagnosis according to DSM‐V. No hearing impairment or ASD	No DLD	8;4–16;0	114	214	
					T1 + 9 months	106	185	7%
					T2 + 9 months	100	158	12.3%
Vermeij et al. (2021)	NL	DLD	Referral to a speech and hearing centre and allocated the tentative diagnosis ‘indicated to have DLD’	×	1;7	185	×	Unclear due to imputation
					T1 + 3 to 21 months	?		
Wadman et al. (2011)	UK	SLI	Attending language unit in the UK and identified with primary speech and language difficulties without neurological difficulties, hearing impairment, autism or general learning disability	TD	7	242		
					16	90	91	42.6%
					17			
Yew and O'Kearney (2015)	Australia	LI	Either (1) positive response to the PEDS language concerns item ‘Do you have any concerns about how your child understands what you say to him/her’, or (2) below the 13th percentile for this cohort on the PPVT, and no hearing impairment, head injury, autism, neurological disorder or epilepsy, and normal IQ. This was validated by (1) need of or use of speech–language pathology services in the past 12 months, and (2) score on the CCC2	TL	4–5	439	2797	Average retention of 88% on follow‐up measurements. Unclear how many participants completed each measurement
					6–7			
					8–9			
					10–12			

*Note*: ^a^Some studies drew data from cohort studies, but they only report on the specific measures and/or time points pertinent to their research.

### 3.2 Findings on the Development of SEF

The 25 studies on SEF focused on different aspects of SEF (Table 2). Therefore, the results will be discussed accordingly, and social and emotional functioning will be discussed separately. Note that some studies investigated both social and emotional functioning and that their results will be discussed in both sections.

**TABLE 2:** Overview of method(s) and results of reviewed literature on SEF.

Study	Cohort	SEF aspect(s)	Instruments	Informant	Covariate(s)	Result
Bao et al. (2016)	Ottawa Language Study	Psychiatric disorders	University of Michigan Composite International Diagnostic Interview	Self	Vocabulary (PPVT), language functioning (TOLD), auditory memory (Goldman–Fristoe–Woodcock Test), Maternal psychological distress (Brief Symptom Inventory), parental SES, educational achievement (Kaufman Test of Educational Achievement; Woodcock Johnson Test of Academic Achievement), NVIQ (WISC), behavioural and social problems (Teacher Report Form), semi‐structured interview (parent‐report), questionnaire on Childhood Sexual Abuse	The probability of having a psychiatric disorder decreased in the LD group, but not in the TD group. LD children were continuously more likely to report lower physical health than TD children
Botting et al. (2016)	MLS	Anxiety	Child Manifest Anxiety Scale—Revised	Self	Gender, NVIQ (WISC/WASI), language composite (CELF‐4)	Anxiety symptoms are stable over time for LI and AMPs, albeit with higher anxiety scores for LI.
		Depression	Short Form Moods and Feelings Questionnaire			Depression in LI decreases to age 17 followed by an increase to 24, also reflected in the % scoring in clinical ranges. Pattern for AMP is stable
Botting and Conti‐Ramsden (2000)	MLS	Behaviour problems	Teacher opinion		Gender, NVIQ (Raven's Matrices), expressive language problems (SLP), receptive language problems (SLP)	Sign. increase in behaviour and social problems for those with expressive and receptive language problems
		Social problems	Rutter Children's Behaviour Questionnaire Harter Perceived Competence Scale for Children	Teacher		
Conti‐Ramsden et al. (2019)	MLS	Peer problems Emotional problems	SDQ peer & emotional problems Rutter Children's Behaviour Questionnaire	Teacher	PIQ (Raven's Matrices; WISC‐III), receptive language (TROG; CELF Word Classes), expressive language (Bus Story Test; CELF Recalling Sentences), pragmatic language (CCC), prosociality (SDQ), parental mental health	Five trajectories: childhood‐onset persistent (increasing) in both (26%) adolescent‐onset in both (16%) consistently low level of problems in both (11%) resolving emotional only (24%) increasing peer problems only (22%)
Forrest et al. (2020)	MCS	Emotion regulation (ER)	Child Social Behaviour Questionnaire	Parent	Spatial ability (BAS II Pattern Construction)	*ER difficulties* decreases from 3–7 years by 1 *Peer problems* decrease from 3 to 5, followed by increases from 5–7, 7–11, and 11–14 All aspects differ significantly from GP At specific time points, the three domains predict one another, but relationships were not reciprocal
		Emotional problems Peer problems	SDQ—emotional problems subscale SDQ—peer problems subscale	Parent (mother)		
Forrest et al. (2018)	MCS	Emotional problems Peer problems/bullying	SDQ emotional problems Short Form Moods and Feelings Questionnaire SDQ peer problems ‘Your Friends’ section of MCS	Parent (mother) Self Teacher Self	SES, Spatial ability (BAS II Pattern Construction), premature birth, gender	Emotional problems increase from age 7 to 14. Also, problem scores of those scoring high at age 7 increase by 7% to age 14 At both ages, the rDLD group was more likely to have slightly raised or high/very high peer problems than the GP group Peer problems mediate part of the relationship between the risk of DLD and emotional problems
Goh et al. (2021)	SCALES	Social–emotional behavioural problems	SDQ	Teacher	SES (IDACI), NVIQ (Wechsler), communication (CCC‐S), Receptive/Expressive One‐Word Picture Vocabulary Tests, receptive grammar (TROG), expressive grammar (SASIT‐E32), Assessment of Comprehension and Expression	Decrease in % borderline for peer problems but increase on all other subscales. Significant differences with TD on all but EP at Y1 and EP and CP at Y3
Jelen et al. (2023)	SCALES	Anxiety	Revised Children's Anxiety and Depression Scale	Self & parent	Language composite (receptive vocabulary (ROWPVT‐4), expressive vocabulary (EOWPVT‐4), sentence repetition (SASIT‐E32), narrative recall and comprehension (ACE), syntax (TROG‐S)), NVIQ (WPPSI‐III), SES (IDACI), COVID‐19, gender, prosociality (SDQ), peer problems (SDQ), social experiences (The Life in Schools Checklist)	Stable anxiety and depression scores between T1 and T2 for both groups. Only in parent reports did children with LD score higher on anxiety and depression. In parent reports but not self‐reports, better language predicted lower anxiety and depression
Levickis et al. (2018)	ELVS	Social–emotional behavioural problems	SDQ	Parent	Expressive language (CELF), receptive language (CELF), core language (CELF), SES (SEIFA), NVIQ (K‐BIT2; WASI), gender, maternal age, maternal vocabulary (Mill Hill Vocabulary Scale), maternal education, maternal mental health, history of speech/language difficulties, SEF (SDQ)	Decrease of total difficulties for the stable DLD group. between 4 and 5y, followed by a slower decrease from 5 to 7. The same pattern for unstable DLD showed an increase from 5 to 7, possibly due to increasing language problems. Consistently decreasing problems for the never‐LD group. Persistent LD has higher conduct, HI, peer and total problem scores at 5 & 7 years compared to unstable LD, who have higher scores than never LD
Lindsay and Dockrell (2012)	×	Behaviour Self‐concept	SDQ Pictorial Scale of Perceived Competence and Social Acceptance for Young Children Self‐Perception Profile of Children/Adolescents/Students	Teacher Self	NVIQ (BAS II Matrices) Expressive narrative (Bus Story) Receptive vocabulary (BPVS II) Grammar (TROG‐2 & CELF‐R Listening to Paragraphs + Recalling Sentences) Reading decoding (NARA‐R &NMRA) Reading comprehension (NARA‐R & NMRA) Spelling (BAS II Spelling),	Total difficulties decrease to 12 years but increase from 12 to 16. Emotional: slow decrease, but drop from 8 to 10 followed by increases of 21% at 12 and 16 HI: steep decrease from 10 to 12, followed by a slow increase to 16 Conduct: increase to 10, decrease to 12, increase to borderline at 16 Peer problems: stable borderline, increase from 12 to 16. Increase in clinical range too Prosocial: increase (positive) to 12, decrease (negative) to 16 Self‐concept: lower than the norm at 16, but improved after leaving school
Mok et al. (2014)	MLS	Peer relations Autistic symptomatology	SDQ Rutter Children's Behaviour Questionnaire Autism Diagnosis Interview—Revised Autism Diagnostic Observation Schedule	Teacher Parent Self	Behaviour (SDQ), PIQ (Raven's Coloured Progressive Matrices; Wechsler), receptive language (TROG), expressive language (Bus Story; CELF Recalling Sentences), reading accuracy (BAS Word Reading; Wechsler), pragmatic language (CCC), Basic Reading & Reading Comprehension, gender, maternal education, household income, placement type	Increase (significant) in peer problems from 8 to 11, and a non‐significant increase from 11 to 16. Four groups: low/no peer problems (22.2%) childhood‐limited problems (12.3%) childhood‐onset persistent problems (39.2%) adolescent‐onset problems (26.3%)
Pickles et al. (2016)	MLS	Conduct problems Hyperactivity	SDQ Rutter Children's Behaviour Questionnaire	Teacher	Expressive language (CELF Recalling Sentences), receptive language (TROG), PIQ (Wechsler), reading ability (Wechsler Basic Reading & Reading Comprehension), prosociality (SDQ)	Five trajectories: Persistent (15%): high levels in both with peak in early adolescence Adolescent‐onset (24%): increase in both from adolescence → normal to GP Childhood‐limited (17%): problems in both that resolved by 16 years Resolving Hyperactivity (16%): no conduct problems, hyperacitivity resolved by age 16 Low/no problems (29%)
Redmond and Rice (2002)	Kansas Longitudinal Study of Morphosyntactic Development	Behaviour problems	Child Behaviour Checklist Teacher Report Form	Parent Teacher	NVIQ (CMMS), language (TOLD‐2)	Social problems remain stable over time Withdrawn and internalizing problems decrease over time At 8, teacher nor parent‐ratings differred SLI group from AM group concerning % in clinical range for social problems Most problems appear at Kindergarten in Withdrawn and Internalizing scales
Roy and Chiat (2014)	×	Social communication	Early Sociocognitive Battery (ESB) Social Responsiveness Scale (SRS)	Self Parent	NVIQ (BAS II), receptive language (CELF‐4), morphosyntax, auditory and expressive language (PLS), behaviour (SDQ, parent‐rated)	Children with LI improved on SDQ scores between T1 and T2, followed by a slight increase to T3 (age 9–11). LI emotional problems and HI increase from T2 to T3, whereas this is not true for NSC‐NL. Those with SCI‐LI showed an increase in problems from T1 to T3. No significant differences between LI and NSC‐NL at T2 and T3
Samson et al. (2020)	Van Den Bedem (2018a)	Social anxiety Emotions Emotion awareness	Somatic Complaint List Social Anxiety Scale for Children—Revised Mood Questionnaire Emotion Awareness Questionnaire	Self Self	PIQ (WISC), emotion communication (Child Alexithymia Measure (CAM)), severity of communication problems (CCC‐2‐NL)	Decrease in social anxiety and somatic complaints for DLD over time, but individual differences remain. These are likely due to differences in emotion understanding, as the initial effects of structural and pragmatic problems in the DLD group disappeared when emotion awareness was taken into consideration
St Clair et al. (2019)	MCS	Emotional problems	SDQ Children's Social Behavior Questionnaire	Parent	Infant temperament, maternal attachment, parental psychological distress, Child–Parent Relationship Scale: Short Form, Child Social Behaviour Questionnaire, vocabulary (BAS‐II Naming Vocabulary), NVIQ (BAS‐II Pattern Construction), Verbal Similarity Subscale	Increasing emotional problems in rDLD and GP, at the same rate. rDLD had higher scores on emotional problems at all time points compared with GP Six subgroups: 1) Low, from age 7 increasing 2) stable very low 3) Low, from age 11 increasing 4) stable average 5) average, from age 11 increasing 6) Very high and reducing Over 55% of rDLD was in subgroup with increasing problems, and they were less likely to be in 2) or 4). 70% of those meeting both rDLD criteria were in 6)
St Clair et al. (2011)	MLS	Behavioural problems Emotional problems	SDQ	Teacher	PIQ, SES, gender, expressive language (Bus Story), receptive grammar (TROG), pragmatic language (CCC‐2), reading abilities (BAS word reading)	Decreasing problems from 7 to 16 on all scales except peer problems, which slowly increased. All problems show an increase from 7y to 8y. All subscales showed significant linear trends (although marginal for emotional problems and peer problems), but not total difficulties
Toseeb et al. (2023)	MLS	Mental health trajectories	SDQ	Parent	Early language and communication environment (ELCE), SES, polygenic scores (incl. MDD, anxiety disorder, ADHD), gender	DLD increase more on emotional problems, conduct and HI than TD DLD are 3x more likely to be in raised groups and have a decreased chance to be in stable low groups Emotional development trajectories: 1) stable low, 2) decreasing within normal range, 3) increasing within normal range, 4) consistently raised HI and conduct development trajectories: 1) stable low, 2) stable within normal, 3) consistently raised
Toseeb et al. (2017)	MLS	Prosociality	SDQ	Self	Expressive language (CELF—Recalling Sentences), receptive language (TROG), receptive language (CELF word classes), gender, NVIQ (Wechsler), Friendship Difficulty Index (Social Emotional Functioning Interview), Community Integration Measure, behaviour (Achenbach checklist)	Stable prosociality over time for LI and AMP, but 2 trajectories for LI: prosocial and moderate prosocial Being in prosocial trajectory is protective of friendship difficulties and community integration at 24y (T3)
Toseeb et al. (2020)	ALSPAC	Prosociality Behaviour	SDQ	Parent	Early language and communication environment (ELCE), SES, language compound score (WOLD), friendships (statements, parent‐report), play (statements, parent‐report), prosociality (SDQ, parent‐report), behaviour (SDQ, parent‐report)	For both DLD and TD better ELCE is associated with better social play and more prosocial behaviour, which are related to fewer externalizing problems. Children with DLD score below TD on social play
Toseeb and St Clair (2020)	MCS	Prosociality	SDQ	Parent	Expressive vocabulary (BAS naming vocabulary), expressive vocabulary (BAS verbal similarities), behaviour (SDQ, parent‐report)	Four trajectories for prosociality: Stable high Stable slightly low Decreasing to slightly low Increasing to high DLD less likely to be in stable high class and more likely to be in stable slightly lowStable‐high DLD had better SDQ compared with those in 2) and 3)DLD maintain lower prosociality across 5–11 years and do not catch up, but they have equal chances of improvement as GP
Tsou et al. (2023)	ALSPAC	Emotion understanding	Emotion Discrimination Task Emotion Identification Task Emotion Attribution Task	Self	Age	DLD and TD performance increases with age on all indices. DLD catch up with TD on all but emotion discrimination task, although no differences were apparent at T1 (less strong improvement)
						DLD increase more in Positive Emotion Identification than TD, as well as in Positive Emotion Attribution
						BUT: baseline difference (T1) between TD and DLD existed for emotion identification and attribution. Negative Emotion performance was already similar to TD at baseline
Van Den Bedem et al. (2018a)	×	Bullying & victimization	Revised Bully/Victim Inventory	Self	PIQ (WISC), severity of communication problems (CCC‐2‐NL, parent), gender, SES, age, emotion understanding	Victimization and bullying behaviour decreased in older children with and without DLD. Emotion understanding increased in this period and led to decreasing feelings of victimization in the DLD group, more so than in the TD group. Bullying was explained by more and increasing feelings of victimization
Van den Bedem et al. (2018b)	Van Den Bedem et al. (2018a)	Depressive symptoms	Child Depression Inventory	Self	PIQ (WISC), severity of communication problems (CCC‐2‐NL, parent), gender, SES, age	Depressive symptoms decreased over time for the DLD group, but not for the TD group
van den Bedem et al. (2019)	Van Den Bedem et al. (2018a)	Empathy Peer relations	Empathy Questionnaire for Children and Adolescents (EmQue‐CA) Best Friend Index (BFI)	Self	PIQ (WISC), severity of communication problems (CCC‐2), SES, gender, age	*Increases*: Affective empathy (DLD & TD) Prosocial motivation (DLD & TD) Cognitive empathy (DLD & TD) Positive friendship features (stronger in DLD) *Decreases*: 1) Negative friendship features (DLD & TD)
Van Den Bedem et al. (2020)	Van Den Bedem et al. (2018a)	Emotional competence Externalizing behaviour	Emotion Expression Questionnaire Child Symptom Inventory ODD scale Instrument of Reactive and Proactive Aggression	Parent Self	PIQ (WISC), SES, severity communication problems (CCC‐2), Children Alexithymia Measure (CAM), gender, age	*Externalizing problems* decreased with age, and emotion recognition increased (both groups!)
Vermeij et al. (2021)	×	Behaviour	Child Behaviour Checklist (CBCL)	Parent	Expressive language (Schlichting), receptive language (Schlichting), receptive vocabulary (Schlichting)	As a group, children with behaviour problems at T1 had fewer problems at T2 (no significance test performed)
Yew and O'Kearney (2015)	LSAC	Emotional problems	SDQ	Parent	Receptive vocabulary (PPVT), NVIQ (WISC‐IV Matrix reasoning), temperament (Short Temperament Scale for Children, parent‐report), quality of peer interactions (parent‐report), maternal depression (Kessler‐6), child Rearing Questionnaire (parent‐report), SES (SEIFA)	Increase in emotional problems between 4 and 11 for LI and TD at the same rate, but LI predicts persistently higher levels of emotional symptoms
Wadman et al. 2011)	MLS	Anxiety	Child Manifest Anxiety Scale—Revised	Self	Receptive language (CELF Word classes), expressive language (CELF Recalling Sentences), PIQ (WISC‐III), gender, educational achievement (GCSE/GNVQ), behaviour (SDQ), bullying (questionnaire)	Stable trend for anxiety in both groups, but SLI had significant higher anxiety scores at 17 compared with TD. SLI improvement on depressive scores, but not TD. At 17, the groups did not differ

#### 3.2.1 Social Functioning

Six studies examined the development of peer relations in children with LD. These studies showed that peer problems in children with LD typically increase between 4 and 16 years (Conti‐Ramsden et al. 2019; Forrest et al. 2020; Mok et al. 2014; St Clair et al. 2011). Conti‐Ramsden et al. (2019) identified five trajectories concerning peer and emotional problems in children with SLI (*n* = 232 at T1). Over 60% of this group had both peer and emotional problems at some point in time. For over 48%, peer problems persisted from childhood into adolescence, and for 16%, problem scores started to increase from adolescence onwards. The remaining 35% of the children with SLI showed consistently little peer problems. Only one study found an improvement in peer relations between 8 and 16 years (Van den Bedem et al. 2019). The sole study looking beyond the age of 16 analysed changes in friendship quality between early childhood and adulthood, that is, age 24 (Howlin et al. 2000). Their entire sample (*n* = 19) had good‐quality friendships in childhood. For 37% of them (*n = 7*), friendship quality was poor at 24 years, and another 6 participants also showed a decline in friendship qualities, albeit not as steep.

Differently, findings on prosociality are generally positive. Three studies report stable or improving prosocial skills, at least to the age of 12 (Eadie et al. 2018; Toseeb et al. 2017; Toseeb and St Clair 2020). Children without LD improve at the same rate during this period, albeit with higher (i.e., better) prosociality scores (Toseeb and St Clair 2020). Note that Lindsay and Dockrell (2012) reported a decrease in prosociality of those with SLI (*n* = 69) from 8 to 12 years. Beyond the age of 12, from 12 to 16 years, the same group of children improved their prosocial abilities. Differently, Toseeb et al. (2017) found a stable trend between 11 and 24 years among both adolescents with and without LI. Within the LI group, two groups could be identified: a prosocial group with scores similar to TD peers and a group with moderate prosociality, that is, scores that were continuously below the TD and prosocial LI groups. One study simultaneously tracked peer relations and prosociality and reported stable improvements from 8 to 16 years in both domains (Van Den Bedem et al. 2019).

Findings on the developmental trajectory of hyperactive and conduct behaviours present a complex picture. Nine studies investigated hyperactivity and conduct behaviours. Difficulties are typically found to decrease in early childhood, approximately between ages 2;6 and 5 or 6 (Eadie et al. 2018; Levickis et al. 2018; Lindsay and Dockrell 2012; Redmond and Rice 2002; Roy and Chiat 2014; Vermeij et al. 2021). This positive trend does not predict equally positive development into late childhood. Between the ages of 6 and 12 hyperactivity and conduct behaviour appear to increase in those with LD (Botting and Conti‐Ramsden 2000; Goh et al. 2021; Roy and Chiat 2014). Some studies find different trends between different ages. Bullying behaviour, which can be considered as conduct behaviour, was found to decrease between 8 and 16 years in children with DLD and remain stable or decrease in those without (Forrest et al. 2018; Van den Bedem et al. 2018b). Similarly, externalizing behaviours such as aggression, opposition defiant disorder (ODD), hyperactivity and conduct problems decreased from 8 to 16 (St Clair et al. 2011; Van Den Bedem et al. 2020). This was preceded by an increase between 7 and 8 years. Differently, Lindsay and Dockrell (2012) reported an increase in problem behaviours into adolescence despite finding improvement between 8 and 12 years like St Clair et al. (2011).

In an attempt to account for the variation reported here, one study identified different developmental trajectories in children with a history of LI (*n* = 164). For 15% (*n* = 25), parents reported persistently high levels of hyperactivity and conduct behaviour (Pickles et al. 2016). There was also a group of children that showed an adolescent onset of both behaviours (*n* = 39), similar to what Lindsay and Dockrell (2012) reported, and a group with childhood‐limited problems (*n* = 27), more in line with St Clair et al. (2011). The remaining children fell either in the resolving hyperactivity group (*n* = 26) or showed no or little problem behaviour (*n* = 47). Unlike what is found in the typical population, conduct behaviour never occurred in isolation in children with (a history of) LI.

#### 3.2.2 Parent Versus Teacher Reports of Social Functioning

Of the studies investigating social functioning, 4 used parent reports and 5 used teacher reports. The studies on depression, anxiety and bullying relied on self‐reports, as participants were adolescents. Of the studies using parent reports, 3 observed a decrease in problem behaviours, while 1 found both an increase and a decrease. Of the 5 studies using teacher reports, 3 noted an increase in problem behaviours, 1 reported a decrease, and 1 found both an increase and a decrease (between different time points). Notably, parent reports are more commonly used to assess children at preschool or early school age (2 to 7 years), whereas teachers report on students aged 7 and above. Thus, according to parents, the social functioning of children with LD improves in early childhood. Teachers observe a decline in children's social functioning starting from age 7, but they may later improve from 10 or 12 years on.

#### 3.2.3 Emotional Functioning

A total of 15 studies investigated emotional abilities in children with LD. Irrespective of the definition of the clinical group, children with LD exhibit more emotional problems than their peers, and these problems increase from childhood to (early) adolescence. Note that the rate of increase is often found to be similar between children with LD and those without (Eadie et al. 2018; Forrest et al. 2020; Yew and O'Kearney 2015; St Clair et al. 2019; Toseeb et al. 2023). Conti‐Ramsden et al. (2019) identified different developmental trajectories for emotional difficulties. More than 60% of children with LD experienced emotional problems at some point between the ages of 7 and 16. Over 25% of them had persistent emotional problems from childhood to adolescence, 16% developed emotional problems from adolescence onwards, and 24% had emotional problems in childhood that resolved in adolescence. The remaining 33% had low levels of problems or no problems throughout childhood and adolescence. A different study also reported the adolescent‐onset trajectory, finding a decrease in emotional difficulties for children with SLI from 8 to 10, followed by an increase from 10 to 16 years (Lindsay and Dockrell 2012). Other studies’ results converge with Conti‐Ramsden et al. resolving the emotional problems trend. Roy and Chiat (2014) found that children with SLI increase in emotional abilities between the ages of 3 and 5. This initial bettering was followed by a decrease to the next measurement (ages 9–11), a trend absent in the control group. Another study reported a decrease in emotional problems in children with SLI from 8 to 16 years (St Clair et al. 2011). Differently, Goh et al. (2021) found increasing problems only for boys with DLD from ages 4–5 to 7–9. It is unclear if this trend persisted or if problems settled after 9 years. One study did not fit into any of Conti‐Ramsden et al.’s trends. Here, emotional difficulties already began to decrease in early childhood, from 4 to 7 years (Levickis et al. 2018). Moreover, the children with and without LD in this sample did not differ from each other in emotional functioning.

Four studies investigated the development of depressive symptoms or (social) anxiety. All used self‐reports to assess these symptoms. In school‐age children with LD, depressive symptoms significantly decrease between 8 and 16 years (Van Den Bedem et al. 2018a; Wadman et al. 2011). This pattern was not found in children without LD, who report stable levels of depressive symptoms over time. Note that between 10 and 16 years, children with LD consistently report significantly higher scores on depressive symptoms than those without (Van Den Bedem et al. 2018a; Wadman et al. 2011). Different from these studies, Jelen et al. (2023) found an increase in self‐reported depressive symptoms for TD and LD solely for girls between the ages of 10–11 and 12–13. There was no effect of time for the TD and LD groups as a whole. Interestingly, this study also included parent reports of the participants’ symptoms of anxiety and depression. Children reported nearly double as many problems as their parents, meaning there was poor agreement between parent and child reports. Also, the effect of time found for girls with LD was not found in parent ratings.

The Manchester Language Study (MLS) collected data on depressive symptoms in adolescents with LD. Their participants reported a decrease in depressive symptoms between 16 and 17 (Botting et al. 2016; Wadman et al. 2011). This decrease is followed by an increase in experiences of depression to 24 years for the LD group, whereas those without LD show a stable trend (Botting et al. 2016). Taking a broader scope, a different study analysed the changes in the probability of having a psychiatric disorder (e.g., depression, dysthymia, bipolar disorder) from adolescence to adulthood in individuals with LD (Bao et al. 2016). Self‐reports highlight a rapid decrease in this probability from 19 to 25 years and a slow decrease from 25 to 31. This pattern was unique to the LD group, as no changes were found for those without LD. Note that the LD group had reported more symptoms of psychiatric disorders at the start than the non‐LD group.

A handful of studies investigated specific aspects of emotional functioning (*n* = 6). Social anxiety (Samson et al. 2020), emotion regulation (Forrest et al. 2020), emotion awareness (Samson et al. 2020), emotion understanding (Samson et al. 2020; Tsou et al. 2023; Van Den Bedem et al. 2020), and victimization (Forrest et al. 2018; Van den Bedem et al. 2018b) were examined. Across childhood and/or adolescence, children with LD improved on all these abilities. They either show similar increases in emotional functioning to children without LD (Van Den Bedem et al. 2020) or they even catch up with their peers (Tsou et al. 2023). An exception is a finding on the development of emotion discrimination abilities, which Tsou et al. explored as part of emotion understanding. Although the LD group performed at a similar level as their peers at the start (ages 2–5), emotion discrimination abilities developed at a slower rate. As a result, the group with language difficulties fell behind at the follow‐up 2 years later. Lastly, two studies assessed victimization in children and adolescents with and without DLD (Forrest et al. 2018; Van Den Bedem et al. 2018b). Feelings of victimization decreased between the ages of 8 and 16 years at the same rate for both DLD and TD children. Only in one study, children with DLD experienced more victimization than their peers (Van den Bedem et al. 2018b).

#### 3.2.4 Parent Versus Teacher Reports of Emotional Functioning

The majority of the studies on general emotional functioning used parent reports (64%). Parents of children with LD are likely to report poorer emotional functioning in their children than parents of non‐language‐impaired children. They do typically report similar development of these abilities: emotional problems in children with LD increase over time, approximately from age 3 to 14. The results of the four studies (36%) employing teacher reports are not as clear‐cut. Both increases and decreases in emotional functioning over time are found (Conti‐Ramsden et al. 2019; Goh et al. 2021; Lindsay and Dockrell 2012; St Clair et al. 2011), as well as the appearance or disappearance of problems until or at a specific age. Such changes typically happen around the transition from childhood to adolescence. This often coincides with the switch from primary school to high school (around the age of 12).

#### 3.2.5 Predictors of Social–Emotional Functioning Over Time

A range of factors were found to predict the development of SEF. When language is concerned, the severity of language and communication problems predicted an increase in social–emotional problems over time in six studies (Goh et al. 2021; Redmond and Rice 2002; Samson et al. 2020; St Clair et al. 2019, 2011).

Four studies highlighted that early pragmatic problems predicted decreasing SEF (Conti‐Ramsden et al. 2019; Samson et al. 2020; St Clair et al. 2011; Van Den Bedem et al. 2020). Others found that specific social factors, namely, prosociality and peer relations, predicted social and emotional development. Conti‐Ramsden et al. (2019) highlighted that children with increasing or persistent peer and emotional problems have lower prosocial skills than those without. Prosociality was also related to more positive and fewer negative friendship qualities (Van den Bedem et al. 2019). Higher levels of prosociality also appear to be protective of developing conduct behaviour (Pickles et al. 2016). In other studies, higher levels of peer problems were associated with more emotional problems (St Clair et al. 2019; Yew and O'Kearney 2015). Two studies on depression highlight the effects of conduct behaviour on SEF. Van Den Bedem (2018b) found that the severity of semantic problems specifically, was related to reports of depressive symptoms. However, this effect was mediated by levels of problems in SEF, namely worry and externalizing behaviour. Effects of SEF‐related difficulties were also reported by Wadman et al. (2011). Only in the LD group did more experiences of bullying and peer problems lead to higher reports of depressive symptoms between 16 and 17 years.

Significant child and environmental factors were gender, socioeconomic status (SES), and non‐verbal IQ (NVIQ) or performance IQ (PIQ). Boys seem more likely to have more social–emotional problems and/or show a decrease in their SEF, trends not seen in girls (Goh et al. 2021; Levickis et al. 2018; Toseeb et al. 2023; Van Den Bedem et al. 2020). This is different for depression and anxiety in children with LD, as girls typically report more such symptoms than boys (Jelen et al. 2023). No such effect is found for adolescents (Botting et al. 2016; Van Den Bedem et al. 2018a; Wadman et al. 2011). Lower SES predicts more social–emotional problems (St Clair et al. 2019; Toseeb et al. 2023; Yew and O'Kearney 2015; Jelen et al. 2023), and in some studies, lower NVIQ or PIQ was associated with lower social–emotional abilities (Goh et al. 2021; Redmond and Rice 2002; St Clair et al. 2019; Van Den Bedem et al. 2020). Another factor that occasionally contributed to social–emotional development was maternal or parental mental health (Levickis et al. 2018; St Clair et al. 2019; Yew and O'Kearney 2015). For instance, parents of children with DLD whose emotional difficulties increased during childhood reported higher levels of psychological distress compared with groups who had low or stable emotional difficulties (St Clair et al. 2019).

### 3.3 Findings on the Development of QoL

QoL was assessed longitudinally by only four studies (Table 3), two of which focused on HRQoL and the other two looked at self‐esteem (Durkin et al. 2017; Eadie et al. 2018; Le et al. 2021; Lindsay et al. 2010). The two studies on HRQoL reported on the same Australian cohort, the ELVS, and analysed the development of QoL in children with and without LD aged between 4 and 9. Using parent reports, Eadie et al. (2018) concluded that group‐level QoL declined over time for DLD (*n* = 70), but not for TD. A more recent study on the ELVS cohort (Le et al. 2021) tried to look for developmental trajectories within the entire cohort and identified three QoL trajectories: (1) stable‐high QoL, (2) reduced and slowly declining QoL, and (3) low and rapidly declining QoL. Of the children identified with low language on at least one measurement, approximately 25% followed the low‐rapid decline trajectory. In contrast, only 4% and 7% of those children with medium and high language abilities were in this trajectory, respectively. Note that out of the three language groups, the children with medium language abilities most often fell into the reduced and slowly declining group. Those children with LL thus seem to be at risk of low and declining QoL, but medium language abilities already put children at risk of poorer QoL.

**TABLE 3:** Overview of method(s) and results of reviewed literature on QoL.

Study	Cohort	QoL aspect(s)	Instrument(s)	Informant	Covariate(s)	Results
Durkin et al. (2017)	MLS	Self‐esteem	Rosenberg Self‐Esteem Scale	Self	Receptive language, expressive language and core language (CELF‐4), NVIQ (Wechsler), gender	For both LI and TD self‐esteem is stable over time. LI report significant lower self‐esteem and social self‐efficacy than TD as well as higher shyness scores
		Shyness	Revised Cheeck and Buss Shyness Scale			
		Social self‐efficacy	Perceived Social Self‐Efficacy Scale			
Eadie et al. (2018)	ELVS	HRQoL	PedsQL	Parent	Expressive language, receptive language (CELF), core language (CELF), SES (SEIFA), gender, maternal vocabulary (Mill Hill Vocabulary Scale), maternal education, history of speech/language difficulties, NVIQ (K‐BIT2; WASI), SEF (SDQ)	QoL scores declined over time for DLD, but not for TD. At 9 years, overall QoL was significantly lower in DLD than TD
						Gender and emotional and peer problems at 4 years explained 23% of variation in QoL ratings in DLD at 9 years
Le et al. (2021)	ELVS	HRQoL	PedsQL	Parent	Language (CELF‐4), SES (SEIFA), gender, Child temperament (Short Temperament Scale for Children—reactivity scale), maternal mental health (Kessler K6), maternal vocabulary (Mill Hill vocabulary scale)	Three trajectories for QoL: (1) stable‐high, (2) reduced with slow decline, (3) low with rapid decline
						60% of those with LL were in (2) or (3), whereas this was less for the TL group
Lindsay et al. (2010)	x	Self‐esteem	Self‐Perception Profile for Adolescents/College Students	Self	Vocabulary (BPVS), oral grammar (TROG), language production (CELF Formulating Sentences), language comprehension (CELF listening to paragraphs), literacy (TOWRE), spelling (BAS II Spelling scale), writing (Wechsler Objective Language Dimensions (WOLD) writing expression scale), NVIQ (BAS II Matrices)	Stable trend from 16 to 17 years in the interpersonal domains (e.g., social acceptance). All other areas improved with moderate effect size (e.g., scholastic competence, global self‐worth). Both boys and girls with LD catch up with their TD peers. Generally, self‐esteem is positive

Both studies on self‐esteem involved participants in adolescence and adulthood. The self‐perceptions of adolescents with SLI show improvements between 16 and 17 years (Lindsay et al. 2010). Specifically, they significantly improved on ratings of scholastic competence, job competence, global self‐worth, physical appearance and athletic competence. Note that males typically rated themselves higher, but this gender effect reached significance only for global self‐worth at age 16 and for physical appearance at age 17. Males and females also both showed lower self‐perceptions on some subscales when compared with the norm. Effect sizes decreased between ages 16 and 17, indicating that the SLI group rated themselves closer to the norm at 17 than at 16. From age 17 to 24 self‐esteem appears to remain stable (Durkin et al. 2017), although those with LD consistently rate themselves as significantly lower on social self‐efficacy and self‐esteem and higher on shyness compared with adolescents with TD.

#### 3.3.1 Predictors of QoL Over Time

For overall QoL, one study found that low language ability predicted lower QoL over time (Le et al. 2021). Another reported that general emotional problems (SDQ), peer problems (SDQ) and gender (being male) together explained 23% of the variation in overall QoL in 9‐year‐old children with DLD (Eadie et al. 2018). Predictors of self‐esteem were only analysed by Durkin et al. (2017). Scores on a language composite at age 17 partially predicted self‐esteem at age 24.

## 4 Discussion

This systematic review of 34 studies focused on the current state of affairs regarding longitudinal research on SEF and QoL in individuals with LD. Here, we will first discuss and compare the results of the development of SEF and change in QoL and their predictors. We will then consider the limitations and implications of this review.

Based on the reviewed literature, it is evident that a single developmental pattern for SEF in children with LD cannot be identified. Nonetheless, certain trends did emerge, which will be discussed separately for social and emotional functioning. In the social domain, the development of various abilities follows divergent trajectories. Peer problems generally increase between 4 and 16 years, whereas prosocial abilities tend to improve over the same period. Hyperactivity and conduct behaviour also exhibit multiple development patterns (e.g., Pickles et al. 2016): for some children with LD, these behaviours are persistent; for others, they are confined to childhood or adolescence. Notably, conduct problems always occur together with hyperactivity. Findings on emotional development are similarly ambiguous. Studies employing the SDQ or Rutter's questionnaire indicate that the emotional functioning of children with LD tends to deteriorate from childhood to adolescence. However, there are also reports indicating more positive emotional development. Conti‐Ramsden et al. (2019) identified different developmental trajectories for emotional (and peer) development, which are corroborated by the results of other studies. A large proportion of children with LD experience emotional problems during childhood, adolescence or both. These predominantly negative findings run counter to findings on the development of specific aspects of emotional functioning, such as social anxiety and emotion regulation, awareness and understanding, which generally improve with age. As these abilities are typically assessed using artificial tasks, outcomes may not accurately reflect real‐life skills, but rather task performance.

Contrary to our expectations, language abilities, whether that is a composite score or a specific language ability, do not consistently predict SEF. Standardized language tests (e.g., CELF) and assessments of pragmatic abilities (e.g., CCC) were predictive in less than half of the studies. Most studies minimally included gender, language abilities, NVIQ and SES as covariates. Male gender, lower NVIQ or PIQ, lower SES and having parents with mental health problems have all been found to predict more and often increasing SEF problems. Some studies examined the reciprocal effects of social and/or emotional abilities on each other. Prosociality in particular appears to be a protective factor against increasing levels of peer and emotional problems, as well as against conduct behaviour (Conti‐Ramsden et al. 2019; Pickles et al. 2016). Importantly, few studies investigated the associations between SEF domains, limiting the generalizability of the findings. The relationship between prosociality and peer problems is particularly complex, as prosociality tends to improve with age while peer problems increase, yet prosocial behaviour is also found to protect against peer problems. A simultaneous assessment of the development of this behaviour is necessary to clarify these paradoxical outcomes.

This review identified only four studies that specifically examined QoL among children with LD. Eadie et al. (2018) reported a group‐level decrease in QoL in children with DLD between 4 and 9 years. Taking the same cohort but a different clinical population, Le et al. (2021) found that a large part of children with LL experienced reduced or low and declining QoL compared with TD children. Self‐esteem was studied in adolescents and young adults with LD and appears to be relatively stable during this period, despite remaining low compared with TD peers (Durkin et al. 2017). Taken together, these studies suggest that QoL decreases in the school age but might reach a plateau in adulthood, at least regarding self‐esteem. Determining what predicts this development of QoL at specific ages remains challenging.

The partly overlapping relationship between SEF and QoL is hard to determine based on comparisons of studies focusing on either dimension. Eadie et al. (2018) administered measures of both SEF (i.e., SDQ) and QoL (i.e., PedsQL) in the same cohort. Their DLD group showed improvements in SEF from age 4 to 9, while their QoL decreased over the same period. Although this trend in SEF is not entirely consistent with other studies included here, it remains noteworthy. SEF also partially predicted QoL: higher scores on peer and emotional problems at 4 years led to lower QoL at 9 years. However, the effect of changes in SEF on change in QoL over time was not analysed, nor the reverse.

There are several plausible explanations for the contrasting results for SEF and QoL. First, it is conceivable that the impact of initial SEF challenges manifests in QoL at a later age, such as during the transition to high school or adolescence when children develop greater self‐awareness and compare themselves more frequently to their peers. Second, as suggested by Pickles et al. (2016) and Conti‐Ramsden et al. (2019), it is likely that children followed different developmental trajectories and that for some SEF and QoL did follow a similar path. Third, the differences between the SDQ and PedsQL may contribute to the observed discrepancies: the SDQ assesses problems across five subscales, namely, hyperactivity/impulsivity, emotional problems, peer problems, conduct behaviour and prosociality, while the PedsQL asks specifically about health‐related QoL in social, emotional, physical and school functioning. The latter two domains specifically distinguish between the PedsQL and the SDQ, possibly also explaining the opposing developments observed by Eadie et al. (2018).

Several notes should be made on the reviewed studies' methodological heterogeneity, given its impact on the generalizability of the results. To start with, the included 34 studies in the review reported on a relatively low number of samples (13/34), with multiple studies reporting on the same sample or cohort, such as the MLS (10/34). Attrition was also high in most studies, especially in those reporting on development across two or more time points. Attrition was typically biased, with participants with more severe language problems and/or lower SES retreating more frequently than those without. This population bias could explain the relative lack of associations between measured covariates and SEF and QoL. If language problems are less severe, the effect of LD on SEF and QoL development may not be as pronounced. Eadie et al. (2018) compared QoL in a subgroup with severe DLD to mild to moderate DLD children. Parents of children with severe DLD solely reported significantly lower school functioning at 9 years and QoL seemed to decline faster in these children.

Another important difference between the studies was the choice of questionnaire respondents. Studies on SEF more frequently used parent than teacher reports. Parent reports typically showed improvement over time, whereas findings on teacher reports varied. Behavioural differences between the home and school environments might also lead to discrepancies between teacher and parent ratings. All QoL studies relied on parent reports. Future use of self‐reports could shed a different light on the matter (e.g., Nicola and Watter 2015).

Third, studies varied widely in their inclusion criteria for the LD group. They typically adhered to the inclusion criteria set by the cohort sample from which they drew their data. However, some only required children to have a low score on one formal language (sub)test (often receptive vocabulary) or a positive response from parents or professionals to a question concerning language problems. Others maintained more strict criteria, such as 1 or 2 SDs below the mean on multiple language tests. This variation leads to inherently different samples of children with LDs across the articles reviewed, which could partly explain the inconsistencies in findings on the development of SEF or QoL.

A last methodological difference concerns the materials and included covariates of the SEF studies. Although the SDQ was the most used measure of SEF, other methods (e.g., Rutter's questionnaire, CBCL, or SRS) were also applied. This diversity complicates the comparison of results. Similarly, drawing conclusions about the effect of predictors on SEF development was confounded by the variation in included predictors. Choices concerning predictors generally depended on sample size, study aims and data availability. It remains uncertain whether a factor with predictive value in one study would have been predictive in another study that did not include this same factor.

This review highlights that the development of SEF in children with LD has received considerable attention in longitudinal research. However, drawing conclusions based on these studies is complicated by the large variety of materials used, choice of respondent, LI criteria, and the inclusion of covariates/predictors. Similarly, making inferences about QoL development in this group should also be done with caution for different reasons. QoL has been understudied by longitudinal designs, and findings on general QoL and specific aspects of mental health do not converge. Longitudinal designs prove very insightful in our comprehension of the development of children with language problems. Yet such designs are rarely used, particularly in QoL research. The QoL of only one cohort, the ELVS, was tracked over time. Despite the relatively large sample size, both studies reporting on this cohort's QoL maintained different criteria for the LD group. This complicates the interpretation and generalization of the studies’ findings. Most studies discussed here did not adhere to DLD diagnosis criteria as requirements for LD group assignment, which could also explain incongruence across findings. Another methodological recommendation concerns the inclusion of predictors: some studies attempt to explain variance by only a limited number of predictors. Language often fails to explain the heterogeneity in results, and findings on other predictors are equally inconclusive. Including SEF as a predictor of QoL could be very insightful, as they appear to be partly related (Eadie et al. 2018). Also, some predictors have not (or only occasionally) been included in estimates of variance. Take for instance children's language background (i.e., multilingualism) and received support (i.e., SLT and/or special education). Both contribute to general and educational development and could aid positive change in SEF and QoL. Also, there is a considerable lack of studies that track both SEF and QoL in the same sample, despite the (preliminary) evidence suggesting associations between the development of both these domains. We argue that longitudinal studies investigating SEF and QoL in the same population with different predictor variables could be useful in clarifying the dynamic interplay between both dimensions.

## 5 Limitations

Several limitations must be acknowledged. Despite creating an inclusive search query, there is always the possibility that relevant studies were missed due to the diversity of labelling in the literature. Also, we opted for a framework that separated social functioning, emotional functioning and QoL, as depicted in Figure 1, to synthesize the literature comprehensively. Although this framework might be different from other classifications used in the literature (e.g., internalizing/externalizing), it best suited the aims of this review.

## Conflicts of Interest

The authors declare no conflicts of interest.

## Data Availability

Data sharing is not applicable to this article as no new data were created or analysed in this study.

## APPENDIX A

### A.1

Search query for *language disorders*:

(‘language impair*’ OR ‘language disabilit*’ OR ‘language defici*’ OR ‘language disorder*’ OR ‘language problem*’ OR ‘low language*’ OR ‘DLD’ OR ‘SLI’ OR ‘LL’)

AND search query for *age*:

(‘child*’ OR ‘pre‐school*’ OR ‘preschool*’ OR ‘toddlers’ OR ‘adolescen*’)

AND search query for *SEF/QoL*:

(‘social–emotional*’ OR ‘socioemotion*’ OR ‘socio‐emotion*’ OR ‘peer relation*’ OR ‘behaviour’ OR ‘behavior*’ OR ‘social*’ OR ‘emotion*’ OR ‘SEB’ OR ‘social cogniti*’ OR ‘anxiety*’ OR ‘anxious’ OR ‘depressi*’ OR ‘internaliz*’ OR ‘internalis*’ OR ‘externaliz*’ OR ‘externalis*’ OR ‘Quality of Life’ OR ‘wellbeing’ OR ‘well‐being’ OR ‘mental health’ OR ‘QoL’ OR ‘HRQOL’ OR ‘quality‐of‐life’)
